# Practical guidance for conducting high-quality and rapid interim analyses in adaptive clinical trials

**DOI:** 10.1186/s12916-025-04362-x

**Published:** 2025-10-01

**Authors:** Helen Mossop, Zoë Walmsley, Nina Wilson, Opeyemi Agbeleye, Michelle Bardgett, Alex Bevin-Nicholls, Matthew Breckons, Michael Cole, Dawn Craig, Munyaradzi Dimairo, Helen Hancock, Martin Law, Andre Lopes, Nurulamin M. Noor, Chizoba Oparah, Philip Pallmann, Julia Phillipson, David S. Robertson, M. Dawn Teare, Katie H. Thomson, Christina Yap, James M. S. Wason

**Affiliations:** 1 Population Health Sciences Institute, Newcastle University, Newcastle Upon Tyne, UK; 2 Newcastle Clinical Trials Unit, Newcastle University, Newcastle Upon Tyne, UK; 3 NIHR Innovation Observatory, Newcastle University, Newcastle Upon Tyne, UK; 4 Clinical Trials Research Unit, Sheffield Centre for Health and Related Research (SCHARR), University of Sheffield, Sheffield, UK; 5 Medical Research Council Biostatistics Unit, University of Cambridge, Cambridge, UK; 6 Royal Papworth Hospital, Cambridge, UK; 7 CRUK Cancer Trials Centre, University College London, London, UK; 8 Department of Medicine, University of Cambridge, Cambridge, UK; 9 Centre for Trials Research, Cardiff University, Cardiff, UK; 10 Clinical Trials and Statistics Unit, The Institute of Cancer Research, London, UK

**Keywords:** Adaptive trial, Interim analysis, Trial conduct

## Abstract

**Background:**

Adaptive designs are increasingly being used in clinical trials within diverse clinical areas. They can offer advantages over traditional non-adaptive approaches, including improved efficiency and patient benefit. The level of improvement observed in practice depends to a large degree on conducting interim analyses (at which adaptations can be made to the trial based on collected data) rapidly and to a high standard.

**Methods:**

The ROBust INterims for adaptive designs (ROBIN) project aimed to identify best practice for conducting high-quality and rapid interim analyses. This was done through evidence synthesis of published work, qualitative research with trial stakeholders working at public sector clinical trials units, engagement with patients and the public, and a meeting of trial stakeholders to discuss findings and agree recommendations.

**Results:**

This paper provides recommendations for teams that conduct adaptive trials about how to ensure interim analyses are done rapidly and to a high standard. We break down recommendations by stage of the trial. We also identify a lack of methodology on how best to involve patients in adaptive trials and related decision-making. A limitation of our recommendations is that the research was mostly focused on UK academic settings, although we believe much of the recommendations are relevant in other countries and to industry-sponsored trials.

**Conclusions:**

When following the recommendations outlined in this paper, the process of planning and executing interim analyses will be smoother; in turn, this will lead to more benefits from using adaptive designs.

**Supplementary Information:**

The online version contains supplementary material available at 10.1186/s12916-025-04362-x.

## Background

Adaptive designs (ADs) [1] have gained prominence in recent years due to the important advantages they offer in the evaluation of interventions. ADs allow the use of accruing patient data to implement prespecified decision rules that modify trial design features of an ongoing trial whilst preserving statistical integrity. Design features that may be modified in an AD include sample size, early stopping, number of arms, inclusion criteria, and allocation ratios, amongst others. Through allowing changes, ADs can (1) improve the statistical power of the trial; (2) reduce the time taken and number of participants required to evaluate treatments; and (3) reduce exposure of trial participants to insufficiently effective, or even harmful, treatments by stopping recruitment early [2].

The use of ADs has increased in recent years [3, 4], in particular, contributing to improving the speed of COVID-19 trials [5]. With increased use, there has been a developing body of literature on practical issues that arise with ADs. This includes papers focused on trial management and data management [6, 7], governance issues in adaptive platform trials [8] and how to appropriately plan the resources required for an adaptive trial [9, 10].

**Fig. 1:**
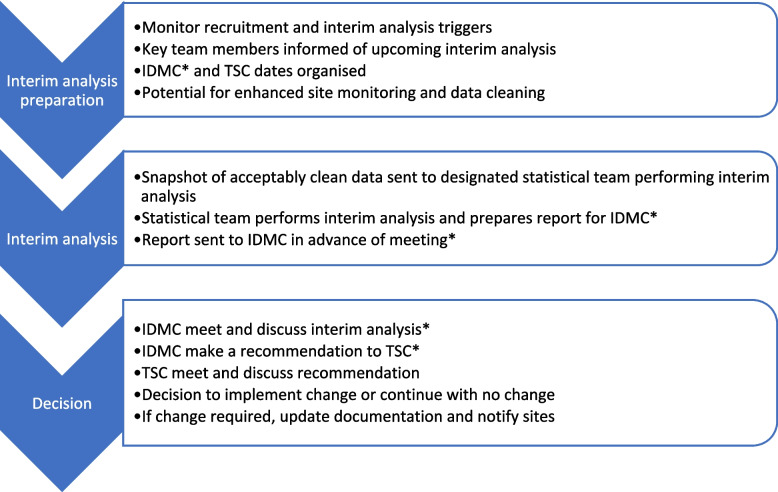
Flow of interim analysis. *Note that, depending on the nature of the design and type of adaptation, the IDMC may agree that they do not need to be informed of all interim analyses, or approve all interim adaptations

A critical component of ADs is interim analysis [11]. An interim analysis is a pre-planned point in the trial, defined either by ‘information time’ (e.g. after 50% of participants have completed follow-up) or calendar time (e.g. every 6 months), where the outcome data collected so far are assessed, and pre-specified adaptations to the trial’s design features are enacted based on these results. For example, in an adaptive platform trial [12], where intervention arms may be added or removed over time, results from an interim analysis may indicate an intervention arm is not showing sufficient promise, leading to it being dropped from the platform. We have provided an overview of the steps involved in conducting an interim analysis in Fig. 1.

For an AD to provide maximal efficiency, the interim analysis must be conducted, and adaptations implemented, quickly, including any required discussions with relevant committees such as Independent Data Monitoring Committees (IDMCs). It also must be done in a high-quality way: the interim analysis must be conducted in a fully pre-specified way and on acceptably ‘clean’ data and in a way that avoids operational biases.

The ROBust INterims for ADs (ROBIN) project aimed to synthesise the current literature, gather stakeholder opinions, and develop clear guidance on improving the quality, speed, and credibility of interim analyses. In this paper, we provide guidance, informed by ROBIN, on improving the speed and quality of interim analyses so that the efficiency of ADs can be maximised in practice. Although focused on phase II onwards and guided by insight from academic settings, many of the recommendations will be relevant to phase I trials and commercially sponsored trials coordinated by Contract Research Organisations (CROs) and pharmaceutical companies.

### The ROBust INterims for ADs project

The ROBIN project was funded by the UK National Institute of Health and Care Research (NIHR) to answer the following research questions:

1. What approaches are being used in the implementation of interim analyses in phase II–IV adaptive clinical trials?
2. What are the facilitators and barriers to interim analyses being high-quality, rapid, and acceptable to stakeholders?

The objectives were to:

1. Investigate and identify existing best practices for conducting high-quality and rapid interim analyses through an evidence synthesis;
2. Augment this literature review via interviews with key stakeholders (including adaptive trial researchers and staff working in UK academic Clinical Trials Units (CTUs)) to understand challenges and how these can be addressed;
3. Get views from patients and the public via a Public Advisory Group (PAG);
4. Develop recommendations for planning interim analyses with key stakeholders; and
5. Prioritise future methodology research required for developing and evaluating further improvements.

The guidance that follows in this paper is formed from the project as a whole, which consists of the following: (1) the evidence synthesis of 61 published papers; (2) interviews with 19 participants (representing data managers, trial managers and statisticians working at public-sector CTUs with varying levels of expertise in ADs), conducted by an expert qualitative researcher (MBr); (3) three meetings of the PAG with researchers involved in ROBIN; (4) a hybrid workshop held in November 2023. A report on the project describes these in more detail, provided as Supplementary Files 1 and 2.

### Best practice guidance

In this section, we provide guidance on conducting high-quality and rapid interim analyses. This is split by stage of the trial in the following subsections. Table 1 presents a high-level summary of the recommendations.

**Table 1:** Overview of recommendations by stage of trial and category

	Trial management	Data management	Trial statistical team	IDMC
Planning stages	• Consider timing and number/frequency of interim analyses • Ensure sufficient resource and capacity to support the use of an adaptive design			• Ensure at least one member has experience with adaptive trial designs • Provide training on the adaptive design for all members (with a Q&A session) • Decide which aspects of interim analysis and trial adaptation require the IDMC and which ones could be automated without their involvement • Consider capacity for attending meetings (potentially at short notice) when choosing committee members • Agree an IDMC charter covering responsibilities, requirements and communication/escalation plans, including when IDMC input to interim analyses is not required • Agree template interim report and consider discussion of simulated interim analysis reports in advance • Arrange meetings in advance where possible, or seek to appoint a larger number of members than usual to ensure quoracy for decision making
Trial setup	• Write key study documents (e.g. protocol and PIS) in a way such that they would not need amending following an interim analysis. Where this is not possible, structure them in a way that would make it straightforward to make anticipated changes • Specify in the protocol expected timeframes for data entry and query resolution at site • Engage with relevant key stakeholders (e.g. regulators and ethics committees) to anticipate risks and plan mitigations • Consider site capacity and capability for delivering the adaptive design • Establish good site engagement and provide sufficient training at SIV on adaptive design features and requirements	• Ensure key data needed for interim analysis is collected in an easy-to-use format (e.g. not requiring complex derivation) which can be easily extracted • Ensure the randomisation system has flexible functionalities to make changes (where required) • Develop a Data Validation Plan to agree data checks to be performed • Build in real-time validation checks to the trial database for key variables • Where required, set up processes for continual data validation and cleaning and how adaptation decisions will be actioned (including any quality control checks)	• Provide examples of individual simulated interim analyses and discuss with key stakeholders • Include, for a range of scenarios, a visualisation of the data used at the interim analysis and what adaptations (if any) the design would recommend • Consider statistical team roles and responsibilities relating to interim analysis (e.g. who will have access to unblinded outcome data, who will stay blinded, who will conduct the analysis, who will do the QC, who will communicate with the IDMC, etc.) • Where there are unblinded and blinded members of the statistical team, ensure SOPs are in place to maintain appropriate firewalls	
Pre-interim analysis	• Prepare documentation for potential adaptations • Monitor timelines for interim analyses and keep all stakeholders up to date • Notify sites of possible changes to the trial and ensure availability to answer queries from site staff	• Ensure an active and continual data cleaning process for key variables with a standardised query resolution process • Consider use of automated software/systems or programs to perform data checks • Perform regular monitoring of data entry and data query resolution and escalate emerging issues early • Consider use of automated data flow processes	• Ensure a SAP is in place (as a minimum for interim analyses) • Develop and validate programs to be used for the interim analysis in advance (statistical quality control process) • Prepare and agree template reports in advance • Consider automating report generation • Prepare a SOP for interim analysis; process, including instructions for how to run pre-prepared programs • Consider a dry run of the interim analysis process using (blinded) trial data • Review communications plan and ensure it is followed, including ensuring appropriate firewalls are in place to restrict access to unblinded data	
During interim analysis	• Maintain good engagement with site teams (e.g. via email notifications and hosting Q&A sessions) • Ensure ‘back-up’ staff are delegated to support onsite monitoring, enter data and respond to data queries	• Prepare database/randomisation system update documents to minimise update time once a decision has been reached • Make sure that any team members who may be required to do testing (UAT, Peer testing, etc.) are aware of pending update and will be available when needed • If a 3rd party supplier is required to make any database or randomisation system updates live, it may be useful to make them aware of timelines	• Train additional staff who can be available at short notice (including to perform QC/validation of analyses where required)	
Post-interim analysis	• Communicate any adaptions to relevant trial team/site staff where applicable/appropriate • Where needed, submit any amendments to trial documents • Deliver training to all stakeholders at site, including staff from supporting departments • Avoid ‘site fatigue’ by offering incentives to, and encouraging competition between, site teams	• Where needed, implement any changes needed to the trial database/randomisation system as a result of the interim analysis • Provide training or guidance to sites on any updates made that effect how the database or randomisation system is used		

*PIS* Participant Information Sheet, *IDMC* Independent Data Monitoring Committee, *SAP* Statistical Analysis Plan, *SOP* Standard Operating Procedures, *SIV* Site Initiation Visit, *UAT* user acceptance testing, *QC* quality control

#### Overarching guidance

In the qualitative research interviews and the in-person workshop, several people made the point that an interim analysis should not be thought of as a single discrete event, but instead as an integral part of the trial as a whole. It is important that the adaptive nature of the trial design is communicated and explained to all parties involved, from design through to the implementation of trial adaptations. The approach to planning and implementing interconnected trial procedures should be done in a way that complements the interim analyses. In subsequent sections, we provide more specific examples of this principle.

Another overarching point identified was the need for capacity building and skills training to allow more complex trials to be designed and delivered. One option for this is through the expansion of targeted training to ensure trial staff have the appropriate skills to conduct ADs. In the UK there are training courses on ADs, but these are predominantly aimed at statisticians to teach how to design and analyse trials that use ADs. It would be impactful to have more short courses focused on the delivery of ADs, aimed at CTUs and research delivery staff. A second option for capacity building is to have more opportunities for shadowing, where less experienced staff can observe experienced staff, potentially in other organisations, and learn from them. Having placements within CTUs and sites that have well-established procedures for ADs would help upskill newer staff.

#### Planning stages

Many of the factors that determine whether interim analyses will be high-quality and rapid can be addressed at the planning stage of the trial. In academic settings, this would include the development of the trial concept and then the process of submitting a grant application to fund the trial. We would first recommend carefully considering whether an AD provides benefit to the trial and participants. ADs are conceptually attractive, but are not always useful [13].

If an AD is useful, then the timing and number/frequency of interim analyses should be carefully planned. Even where it is beneficial to use an AD, it may be that most of the benefit comes from using a relatively straightforward AD with fewer interim analyses. Doing unnecessarily complex ADs may make it more difficult to ensure interim analyses are done to a high standard and quickly. The choice of design may influence the timing of the interim analyses. If an interim analysis is planned early in the trial’s recruitment process, it may mean there is less time to undertake the preparations described in subsequent subsections (as well as outcome data being more variable, potentially impacting credibility). Having predictable timing of interim analyses, such as basing them on calendar-time rather than information-time may help with scheduling issues that are described later. However, if recruitment rates are uncertain, scheduling interim analyses using calendar-time would introduce more variability in sample sizes available at interim analyses, affecting statistical properties of the design.

The importance of simulation studies has been covered in previous work [1, 14]. However, we also recommend that examples of individual simulated interim analyses are discussed with key stakeholders such as clinicians and IDMCs. This would include a visualisation of the data used at the interim analysis and what adaptation(s) (if any) the design would recommend. This allows discussion of potential disagreements with the AD’s recommended adaptations that may have otherwise arisen during an actual interim analysis, causing delays. Such discussion may then lead to refinements in the AD.

During planning stages, it is important to ensure that there are adequate resources for supporting the trial. In particular, the resources required will be affected if the trial uses an AD. The Costing Adaptive Trials (CAT) project investigated the additional staff resource required to support an AD through asking several CTUs to undertake a mock costing exercise. It found that there was variability in the extra amount estimated to be required between different CTUs and for different ADs [9]. The overall conclusion was that the median estimated additional cost was moderate for data management and statistical staff and low for other categories of staff.

Guidance from the CAT project was published [10] to help researchers, funders, and sponsors think through the resources required for an AD. Some aspects are also relevant to conducting high-quality and rapid interim analyses. Since the timing of additional work required for interim analyses might be unpredictable, having flexibility within a larger team is desirable. The availability of an agile, adequately resourced CTU with trained in-house staff is pivotal to delivering high-quality ADs. In some examples from published literature, staffing shortages were mitigated by partnering with a healthcare staffing company (e.g. to hire off-site data entry specialists [15]). We would recommend that substantial senior-level project management oversight is included to deal with the higher level of operational complexity and larger staffing numbers.

Some funding bodies are very supportive of adaptive trial designs and are aware that resources required to support them may be higher. In this case, we would recommend that all costs (particularly for site activities and data management, which may be overlooked) are accounted for in the grant application. Other funding bodies may not be aware of resources required for ADs and generate pressure to reduce costs. We would recommend this is resisted: an under-funded adaptive trial will likely cause delays, put unacceptable workload strains on staff [16] and/or threaten the quality of the interim analysis. In particular, it is impossible to simultaneously prioritise cost reductions, speed, and quality, and so under-funded ADs will risk compromising at least one of the other two criteria.

#### Trial setup

If the trial is funded, then the next phase is the setup. This consists of several interconnected aspects, including (1) development and finalisation of the trial protocol; (2) developing Case Report Forms (CRFs) and setting up the database and randomisation systems; (3) applying for ethical, governance, and regulatory approvals; (4) setting up oversight committees; (5) finalisation of participant-facing materials such as the Participant Information Sheet (PIS); and (6) site selection and setup. While these aspects relate to all randomised trials, carefully considering how they may lead to barriers and facilitators of quick, high-quality interim analysis in ADs is important.

As recommended by the United States Food and Drug Administration (FDA) guidance on ADs [17], the trial protocol should fully describe all aspects of the design. Depending on the complexity of the AD used, it may be possible to write the protocol in a way such that it does not need amending following an interim analysis, regardless of the adaptations made. If this is possible, we would recommend doing so as it will speed up the implementation of changes made due to avoiding protocol amendments being necessary. For more complex ADs, such as adaptive platform trials, it may not be possible to do this. Instead, it is recommended to structure the protocol in a way that makes it straightforward to make anticipated changes such as adding and dropping arms, typically using a ‘master protocol’ framework. Further details are provided in Schiavone et al. [6].

A critical factor in successfully conducting high-quality and quick interim analyses is having the required data recorded in the trial database and the ability to efficiently check and clean the data. Thus, the trial database plays a crucial role in an AD. We would recommend the use of tools like Data Validation Plans (more details are provided in Supplementary File 3) which help prioritise which variables are critical to interim analyses and provide a series of checks that allow flagging potentially incorrect or inconsistent data. Having a process to clean the data in an ongoing manner, especially the data for interim analyses, avoids delay in conducting the interim analysis. We would also recommend, where possible, key data required for interim analyses are collected in an easy-to-use format to avoid the need for complex programming being required (e.g. for calculating treatment adherence). Structuring the database in such a way to allow easy extraction of key data needed for interim analyses could also be beneficial. For ADs where the randomisation may change as a consequence of the interim analysis (e.g. response adaptive randomisation or adaptive platform trials) the randomisation system used should be designed to be flexible to cope with possible changes; otherwise, there will be delays in implementing results of interim analyses. If outsourcing randomisation to a randomisation service, they should be made fully aware of the possible changes as early as possible.

Regulatory engagement may be needed prior to the initiation of the trial if its results will be used in a regulatory submission. For this, there is a need for those with regulatory and governance responsibility to grasp the principles of the AD used. It will be important to anticipate any risks that the AD will cause with regard to operational or statistical biases and plan mitigations for them. This will be true also for ethical approvals and the impact that the AD may have on study participants: as an example, it will be important to consider what happens to participants who are on an arm that is dropped at an interim analysis. Anticipating risks and planning mitigations will hopefully avoid the need for repeatedly applying for approvals, which can considerably slow down the trial.

IDMCs play a crucial role in clinical trials [18], ensuring that the trial remains ethical to continue. Oversight of ADs is typically more complex than that of non-adaptive trials [19]. For some ADs where there are very frequent interim analyses, it may be agreed with the IDMC that not every interim analysis requires discussion. More discussion about this is in the ‘Considerations for specific ADs’ section. However, it is more typical that all results of interim analyses will be presented to the IDMC together with the recommendation for adaptations to be made (or not). The IDMC may agree with the recommendation or feel they need to recommend an alternative course of action. In most cases, we would strongly recommend the IDMC is briefed that they should follow the pre-specified design, unless there is compelling and clearly documented reason not to do so (e.g. strong ethical grounds). If the statistical properties of the AD assume the design is strictly followed, then deviating from the design recommendations may have negative impacts on the evidence produced by the trial (e.g. inflated type I error rate). This is another reason that ensuring all IDMC members are fully aware of the design and its implications, and support it, is important (see the ‘Planning stages’ section). Some ADs, such as group-sequential designs using non-binding stopping rules, are more robust to deviations from the design recommendations. In this case, there are fewer negative implications of the IDMC recommending to deviate from the recommended adaptation.

The resulting IDMC recommendation may then be further discussed by sponsors/funders and other committees (e.g. in the UK, publicly funded trials often have a Trial Steering Committee (TSC) [20]) and finally any changes implemented. This process can be highly variable in length, and various parts can be shortened with the use of processes and tools such as AD-specific communication/escalation plans. It is particularly important to plan the process that will occur if there is a disagreement between the different committees about a recommended adaptation and who has the final decision.

IDMCs are typically formed during the setup phase of the trial. When identifying IDMC members, it is recommended that at least one member is experienced with ADs and that training is provided for all members [21]. This training could include reviewing a simulated interim analysis as is recommended in the ‘Planning stages’ section. If the AD is such that it is difficult to forecast the interim analysis date, then it is recommended that the trial seek to appoint a larger number of members (e.g. 4–5 instead of 3) so that quorate meetings can be held at relatively short notice. Additionally, selecting members with overlapping regular weekly availability can facilitate planning by allowing interim analyses to be scheduled more flexibly. In our experience, choosing members who have very restrictive calendars makes it difficult to schedule meetings. If ADs are designed in a way where the interim analysis timing can be predicted more precisely, then having meetings organised several months in advance is recommended.

Various aids to ensure smooth communications and decision-making have been recommended and may be worth considering in order to support fast interim analyses. Sanchez-Kam et al. [21] emphasise the importance of committee charters to spell out responsibilities and requirements for meetings to be quorate. They also recommend use of an operations plan that details data transfer arrangements, who is undertaking interim analysis and information that the interim analysis report will contain/who has access together with rules and procedures for making adaptations. Similarly, a communications plan was used in some trials [18–20], which sets out how the IDMC and the sponsor/funder should liaise.

In preparation for launching the trial at sites, we would recommend that the PIS and other site documents are prepared in a way that they can be quickly modified for all possible adaptations. As an example, for an adaptive platform trial, the trial team may choose to prepare a ‘master’ PIS containing the invitation to participate, overarching details of what is involved in the trial and supporting information such as safeguarding of confidentiality and details of funding. Additional, shorter, and arm-specific PISs that describe possible adaptations could then be prepared. These could be submitted for regulatory approval at trial outset, so they are ready to use when/if required, and amended separately from the master PIS if necessary.

It is important that site personnel are aware of the implications of interim analyses on their role. Extensive site engagement and training may help with ensuring timely data collection, input, and query-checking. Feasibility questionnaires sent to sites should assess the capacity of the site team to accommodate frequent monitoring visits (which may be more likely in trials with ADs), enter data, and respond to data queries in a timely manner, ensuring ongoing data quality and hence more rapid interim analyses.

#### Pre interim analysis

The period of the trial between starting recruitment and the first interim analysis is critical in ensuring that the interim analysis is done well.

With priority on the key variables needed for the interim analysis, data cleaning should be an active and continual process. Previous work has recommended making this process more efficient through the use of risk-based streamlined cleaning [22, 23]. A specific study using an adaptive seamless phase 2/3 design [24] implemented a process whereby sites had up to 48 h from the study visit to enter data, with automated review and validation daily. Where queries arose, having a standardised data resolution process was useful to ensure these were resolved. We would recommend similar requirements be specified in AD protocols and also that there is a dedicated point of contact within the central trial management team with whom site staff can address queries.

In some trials, automated data flow processes have been set up: these use integrated systems linking data from the collection system to the adaptive analysis program [24, 25]. This would be worth the investment in time and resources if there are multiple interim analyses (such as in response adaptive randomisation designs with frequent updating of the randomisation ratio or group-sequential designs with many interim analyses), but may be excessive for a study with fewer interim analyses.

Prior to the first interim analysis, a Statistical Analysis Plan (SAP) that covers the interim analysis should be in place. We would recommend that this SAP also includes detail of the final analysis; in particular, how the scope of adaptations affects the final analysis (for example, use of unbiased or bias-adjusted estimation methods [26]). In some cases, such as when the interim analysis and final analysis will be undertaken by separate statistical teams, it may be preferable to have separate SAPs for the interim analysis and the final analysis. In all cases, it should be clear who is responsible for the interim analysis, including who will be blinded and unblinded, and any steps to ensure the interim analysis does not cause biases to the final analysis described. This would include having appropriate processes and firewalls in place to restrict access to unblinded trial data.

There are a variety of preparatory steps that could be undertaken to make the actual interim analysis a smoother process. Dey and Pyle [27] recommend a standard operating procedure for interim analyses, comprising stakeholder roles and timelines for data collection, programme development, validation, testing, and execution. In particular, these should be discussed with IDMC members so that expectations and timelines can be agreed.

A step further would be to have a full dry run of an interim analysis in advance of the first actual one. Ideally, this would use real trial data, with treatment assignments available to the unblinded interim analysis team to ensure they gain experience with the full process, while any blinded statisticians remain shielded from unblinding information. By ensuring data cleaning processes and statistical analysis programmes are in place, issues that could cause delays could be identified in advance and addressed. However, a full dry run would also cause an increased burden on trial staff and oversight committee members: we would suggest this only for cases where it is critical to get the first interim analysis done very quickly. A less burdensome, but still useful process, is for the statistical team to prepare code based on an early snapshot of blinded data. If the code is prepared in a way that can easily be rerun on the actual data (e.g. through use of semi-automated reports based on literate programming e.g. with RMarkdown, markstat or Stata (e.g. putdocx)), it can speed up analysis times considerably.

For high-quality interim analyses, we recommend independent quality control (QC) check of the interim analysis by another statistician. It would be important to have a dedicated statistician identified in advance and who is provided sufficient training on the trial, data collection and analysis methods to undertake this role efficiently. A risk-proportionate approach to validation should be considered [28]. Where statistical code is written in advance of the interim analysis taking place, the QC check/validation of the code could be undertaken at this point to reduce/remove the level of QC required at the interim analysis stage.

Where code has been prepared in advance, a trial-specific standard operating procedure could be written to describe the interim analysis process and give instructions on how to run the programs: this would be particularly beneficial if the designated trial statistician is not available.

#### During interim analysis

With the advanced preparation described in previous sections, the actual interim analysis should be a smooth process. However, we would recommend some steps are taken during this time to ensure adaptations are implemented quickly.

Trial sites should be notified in advance that there is an interim analysis underway. They can begin to prepare for making changes quickly and effectively, or at least highlight in advance any issues with implementing changes. Trial managers should help support smooth and efficient implementation. Effective engagement strategies could include timely email notifications, training videos, and drop-in Q&A sessions, which allow site teams to clarify any concerns.

The interim analysis process can be a time of high workload for trial staff and it is therefore important that there is frequent monitoring and prioritising of workload undertaken at a management level as well as by researchers themselves. Having additional trained staff to support and undertake interim analyses, as well as to enter trial data, respond to data queries and accommodate onsite monitoring visits, who may have availability at short notice, is ideal but easier to realise in larger CTUs than in small organisations. Planning ahead will hopefully mean staff are better equipped to cope with the additional pressures that arise during interim analyses.

#### Remaining period of trial

Once the current interim analysis is complete, it is recommended to learn from the entire process and update any procedures described in the ‘Pre-interim analysis’ section. The urgency of this will depend on whether there are additional interim analyses planned in the current trial. Even if not, it will be important to ensure any lessons learnt are recorded for future adaptive trials.

During the course of an adaptive trial with multiple interim analyses, ‘site fatigue’ may set in, where the regular periods of increased workload lead to issues with staff engagement. Discussion amongst ROBIN stakeholders led to the recommendation that consistent engagement with site staff be conducted; incentivisation through competition and rewards was suggested by stakeholders as strategies to maintain momentum. Likewise, there may be similar issues with CTU staff caused by workload. Previous work has identified that regular workload prioritisation across the team is important to prevent overwork and burnout [6].

It is also important to review membership of oversight committees and ensure that a sufficient range of expertise is available as the trial continues. Over long-term adaptive trials such as platforms, there may be issues caused by the relatively high burden on oversight committees. Implementing limited terms and refreshing committees with new members may help ensure that interim analysis results continue to be assessed and adaptations implemented quickly and to a high standard.

#### Dissemination of trial procedures

During the project, we identified some papers that provided an overview of how real ADs were implemented. These serve as useful case studies to inform others about best practice. We would recommend that researchers who successfully implement a high-quality and rapid interim analysis publish their procedures and experiences for others to learn from. It is typically not possible for sufficient detail to be provided in a paper reporting trial results, so these details may be better described in companion papers or supplementary materials. We would recommend providing as many specifics as possible; for example, rather than simply noting that an efficient data cleaning platform was used, authors should detail the platform’s setup and processes to ensure reproducibility as well as dissemination of positive and negative lessons learnt.

Having more general literature on implementing interim analyses, including examples of data validation plans, Gantt charts for scheduling, and Standard Operating Procedures would help others to improve their processes. In addition, as noted in the ‘Overarching guidance’ section, we identified the need for mentorship and shadowing opportunities within and between organisations such as CTUs to support new trial staff to develop their skills.

#### Considerations for specific ADs

The above subsections are general considerations for ADs; however, not all trial adaptations and implications should be treated in the same way—as such, there are some considerations for specific ADs.

For some types of ADs, it may be less important that the interim analysis is done rapidly. An example is sample size re-estimation when the interim analysis is done well before the originally planned sample size. Trial participants recruited during the period when the interim analysis is being done will not be treated differently than in the situation where the interim analysis has been done quicker.

In some trials using response adaptive randomisation, allocations are adjusted at very frequent intervals. If allocation to different arms is changing frequently, then it may be agreed with the IDMC that oversight is not needed for each such adaptation if not implemented simultaneously with other trial adaptations (e.g. futility early stopping). As previously mentioned, this may mean that the process can effectively be fully automated. In that case, we would recommend the first interim analysis is reviewed more thoroughly and the IDMC receives frequent updates about adaptations so that they can raise any concerns that would then warrant a meeting. Examples of such a situation include when the allocation ratio has become sufficiently extreme to warrant considering early stopping of an arm.

## Discussion

ADs have become more prominent and widely used in the last decade. A key component of ADs is the interim analyses that allow accruing data to inform decisions to trigger changes to the way the trial is being run. Whether an AD provides benefit is strongly affected by two key factors: (a) whether interim analyses are done in a high-quality way such that decisions can be relied upon and (b) how long it takes to conduct the interim analyses and implement adaptation decisions. The ROBIN project was established to investigate methods, procedures, and tools that could help ensure interim analyses are conducted to a high standard and quickly.

This paper builds upon the findings from evidence synthesis and qualitative research components of the ROBIN project. Here, we have sought to summarise our recommendations to trial teams who are starting and running adaptive trials. We have divided guidance into different stages of the trial from conceptualisation and setup to post-interim analysis. Table 1 provides a summary of our recommendations and a modified form is provided as a checklist in Supplementary file S4.

During the project, we identified some areas for further methodological work. One important but under-researched area is the role of Patient and Public Involvement & Engagement (PPIE) and how to conduct meaningful PPIE during the interim analysis process within adaptive trials. The ROBIN PAG suggested that more could be done to involve PPIE partners in key stages such as decision-making following interim analyses, but how it should be done is largely unclear and potentially challenging. We believe considering the potential opportunities and barriers to including the perspective of the patient in high-quality and rapid interim analyses is a fruitful area for future co-development work.

Related to this, the question of communication with trial participants was mentioned in our stakeholder discussions. The interim analysis may have a direct impact on participants, especially if treatment arms might be dropped in the trial. Based on our evidence synthesis and qualitative research, there appears to be limited knowledge on what level of communication should be adopted in the immediate period following an interim analysis and, in particular, what should be communicated to patients already recruited and potentially still in follow-up. We believe this is another area that would warrant future work.

Although our research was focused on phase II onwards, many of our recommendations are relevant to phase I trials. However, phase I trials have unique features, such as more frequent interim analyses after small cohorts to assess safety, inform dose recommendations, and support key adaptations. They also typically have direct involvement of unblinded sponsor personnel. These trials often require recruitment to be paused while awaiting sufficient data maturity, which further highlights the need for timely and high-quality interim analyses, particularly in model-based designs.

The qualitative research and hybrid workshop involved individuals who work predominantly on academic clinical trials. We acknowledge that some of our recommendations may not be as germane to commercially sponsored trials that are coordinated by CROs or pharmaceutical companies.

We also identified a need for more real trial examples with sufficient amounts of detail on how the interim analyses were operationalised. We would recommend that for future trials, CTUs published detailed case studies outlining the practical challenges they faced and how they overcame them. One particularly valuable area for such detailed case studies would be the automation of interim analyses—especially in trials with a large number of planned analyses, such as those using response adaptive randomisation. Sharing these operational insights would help build a stronger evidence base and support others in implementing similar designs.

This guidance identifies some issues arising from ADs in general. Some adaptive trials put more burden on oversight committees. Anecdotally, it is rarely easy to find suitable members for adaptive trial oversight committees (e.g. IDMCs and TSCs) and individuals with specialist skills are often overloaded with invitations. There is (at least in academic trials) no direct monetary reward for being involved in these committees and it is seen as part of ‘good academic citizenship’. If ADs are to further increase in use, fresh thinking on how to incentivise being part of these committees would be welcomed.

Consideration of the workforce issues that are exacerbated by adaptive trials is important. Site and trial staff involved in adaptive trials may find the waves of high workload hard to cope with. It is important to ensure that there are adequate staffing resources for more complex designs [10] and that staff have appropriate training opportunities.

There is a need to carefully consider SAPs for ADs, especially what should be included in the version finalised prior to the first interim analysis. A recently funded MRC/NIHR APT-SAP project (UKRI615/APP42050) is focused on developing international consensus-driven guidance for SAPs in adaptive and platform designs.

We hope that the findings from the ROBIN project will encourage increased appropriate use of ADs in a way that provides maximum benefit to all trial stakeholders. Through this, ADs can maximise their impact on improving patient outcomes and the efficiency of research.

## Supplementary Information

Supplementary Material 1: ROBust INterims for adaptive designs (ROBIN): developing best practice for high-quality and speedy interim analyses in Phase II-IV trials.

Supplementary Material 2: ROBust INterims for adaptive designs (ROBIN): Patient and Public Involvement (PPI) Report.

Supplementary Material 3: Appendix – Data Management and Validation Plans.

Supplementary Material 4: Role-Specific Checklists for Interim Analyses in Adaptive Trials.

## Data Availability

No datasets were generated or analysed during the current study.

## Abbreviations

AD: Adaptive design
CAT: Costing Adaptive Trials project
CRO: Contract Research Organisation
CRF: Case Report Form
CTU: Clinical Trials Unit
FDA: United States Food and Drug Administration
IDMC: Independent Data Monitoring Committee
NIHR: National Institute of Health and Care Research
PAG: Public Advisory Group
PIS: Participant Information Sheet
PPIE: Patient and Public Involvement & Engagement
ROBIN: ROBust INterims for adaptive designs project
SAP: Statistical analysis plan
TSC: Trial Steering Committee
