# Supplementary material for: Practical guidance for conducting high-quality and rapid interim analyses in adaptive clinical trials
Source: BMC Med. 2025 Oct 1;23:528. doi: 10.1186/s12916-025-04362-x (PMC12487222; doi:10.1186/s12916-025-04362-x)
Supplement: Supplementary file 1 — Supplementary Material 1: ROBust INterims for adaptive designs (ROBIN): developing best practice for high-quality and speedy interim analyses in Phase II-IV trials. [file 12916_2025_4362_MOESM1_ESM.docx]

1. **Title of Project**

ROBust INterims for adaptive designs (ROBIN): developing best practice for high-quality and speedy interim analyses in Phase II-IV trials

1. **Abstract**

Background

Adaptive designs are increasingly being used in practice within diverse clinical areas. Adaptive designs offer advantages over traditional non-adaptive approaches, including improved efficiency and patient benefit. Actual improvements observed in practice are affected by whether interim analyses (at which adaptations can be made to the trial on the basis of collected data) are done quickly and to a high standard.

Methods

The ROBust INterims for adaptive designs (ROBIN) project had the aims of identifying best practices for conducting high-quality and speedy interim analyses. This was done through several strands of work:

1) An evidence synthesis of published work that proposed methods, procedures or tools to help make interim analyses quicker and higher-quality;

2) Qualitative research of trial stakeholders to investigate current approaches and barriers to adoption of new approaches;

3) Engagement with patients and the public via a Public Advisory Group (PAG);

4) Development of practical guidance and recommendations for future research.

Results

For the evidence synthesis, a total of 61 published papers were included for full-text extraction. Interviews with 19 trial stakeholders were conducted by a trained qualitative researcher. A PAG was formed that consisted of 7 members, which met 3 times.

A stakeholder workshop was held in November 2023, where findings from the project were discussed to agree key points. Some key recommendations for making interim analyses high-quality and speedy based on all the research included:

1) The interim analyses should be considered as a process rather than single events during the trial; careful consideration should be made about how all aspects of the trial support the conduct of quick and high-quality interim analyses.

2) Ongoing data cleaning is important, with priority on data to be used in the interim analysis;

3) Having a full dry run of an interim analysis in advance of the first official one may identify potential issues, allowing for necessary refinements to be made in advance;

4) It is important to properly resource adaptive trials, otherwise there is a risk of delay to interim analyses or there being mistakes made;

5) There is very limited information about how to properly involve patients in interim analyses.

Conclusions

Trials using adaptive designs can maximise their benefit through ensuring that interim analyses are conducted quickly and to a high standard. This work has summarised current best practice for this although further work is motivated, including how to best involve patients in the interim analysis process.

1. **Introduction**

With the rising costs of trials(1,2) and the urgent need to answer crucial research questions, adaptive designs(3) (ADs) have gained prominence in recent years. ADs allow the use of accruing patient outcome data to guide pre-specified changes to design features (such as sample size, number of arms or overall duration) of an ongoing trial whilst controlling statistical integrity. This can: improve the statistical power of the trial; reduce the time taken and number of participants required to evaluate treatments; and reduce exposure of trial participants to insufficiently effective, or even harmful, treatments by stopping recruitment early(4). Use of ADs is increasing in recent years(5,6); in particular they notably improved the speed of COVID-19 trials(7).

ADs involve planned interim analyses, where the outcome data are assessed during the trial, and pre-specified adaptations to the trial enacted based on these results. For example, in an adaptive platform trial(8), where intervention arms may be added or removed over time, results from an interim analysis may indicate an intervention arm is not showing sufficient promise, leading it to being dropped from the platform.

For an AD to provide the most benefit, a number of things must be true:

1. the outcome measure on which any adaption is based must be observed sufficiently soon compared to recruitment length(9,10);
2. the interim analysis must be conducted, and adaptations implemented, quickly, including discussions with and recommendations from Independent Data Monitoring Committees (IDMCs);
3. the accumulating comparative data must not be inappropriately disclosed;
4. the interim analyses must be conducted on high-quality ‘clean’ data.

If a) or b) are not true, the AD provides lower efficiency and trial participant benefit; if c) is not true, the credibility of the trial and its findings is damaged; and if d) is not true, the risk the adaptation will have severe negative consequences on decision-making such as dropping an effective arm is increased.

Guidance on improving the quality of interim analyses and tools and tips to increase the speed of undertaking interim analyses would be highly beneficial to the efficiency provided by ADs. The ROBust INterims for ADs (ROBIN) project was set up to synthesise the current literature, gather stakeholder opinions, and develop clear guidance on improving the quality, speed and acceptability of interim analyses.

1. **Methods**

4.1 Aims and Objectives

The research questions that the ROBIN project sought to answer were:

1. What approaches are being used in the implementation of interim analyses in phase II-IV adaptive clinical trials;
2. what are the facilitators and barriers to interim analyses being high quality, speedy and

acceptable?

The objectives were to:

1. Investigate and identify previous best practices for conducting high-quality and speedy interim analyses through an evidence synthesis;
2. Augment this literature review via interviews with key stakeholders (including adaptive trial researchers and staff working in UK academic Clinical Trials Units (CTUs)) to understand challenges and how these can be addressed;
3. Get views from patients and the public via a Public Advisory Group (PAG);
4. Develop recommendations for planning interim analyses with key stakeholders;
5. Prioritise future methodology research required for developing and evaluating further improvements.

4.2 Evidence Synthesis

A scoping review methodology was employed to identify existing literature highlighting best practice with regards interim analyses. Medline and Embase databases were searched for studies published between January 2005 and May 2023. Following the removal of duplicates, screening at title, abstract and full-text level was undertaken in duplicate, and data extraction was completed by one reviewer and checked in full by another.

Papers were considered if they discussed adaptive trial methodological approaches (phases II-IV) in any clinical population and condition. A narrative synthesis focused on the following outcomes was conducted: trial committee processes, project/trial management, data management, implementation of interim decisions, design and statistical considerations, and patient and public involvement and engagement (PPIE).

4.3 Qualitative Research

An invitation was circulated via professional and personal networks inviting people to take part in an interview about adaptive trials. The inclusion criteria were broad; that participants were involved with the design, running or analysis of clinical trials. A purposive sampling strategy was intended to ensure a mix of job roles and levels of experience in adaptive trials and these details were requested upon expression of interest in participation. Participants were offered a £25 voucher as an incentive and given a choice of face-to-face interviews (if local) or using an online platform.

A flexible topic guide was developed with input from the study team asking about professional background, perspectives on ADs and thoughts on best practices in interim analysis. This was reviewed at regular intervals throughout the interview process so that revisions could allow further exploration of issues arising from data. Interviews were digitally recorded and transcribed verbatim.

Transcripts were imported into NVivo and data were coded using a mixture of deductive and inductive techniques. This process was informed by the categories developed during the evidence synthesis work. Coded data was examined for themes and, while led by MB, data were regularly presented and discussed at project team meetings as well as with the PAG and the wider stakeholder meeting.

4.4 Public Advisory Group

A group of seven public contributors - the ROBIN PAG - steered the design and delivery of the project, ensuring that we focused on not only the quality and speed of interim analyses, but also, crucially, their acceptability from a public perspective. A PPIE Lead (ABN) facilitated their involvement and offered training and support, including through interactive activities such as role plays to help communicate the complex topic.

The PAG comprised public contributors with a range of lived experience. In early discussions, the PAG helped us to consider the impact of interim analyses and resulting changes to a trial on participants, and suggested exploring how PPIE has been embedded in the interim analysis process previously. We made changes to the evidence synthesis plans and interview topic guides, so that we could collect any data about these issues.

4.5 Ethical Approval

The project was reviewed and classified as low risk by Newcastle University’s ethics team due to it not involving patients (reference number 30889/2022).

1. **Results and Conclusion**

A summary of results is provided in subsequent subsections, but detailed results are to be published in a series of papers.

5.1 Evidence Synthesis Findings

In total, 5,726 abstracts were assessed for inclusion. Sixty-one papers representing 56 unique studies were deemed eligible for inclusion in the scoping review. Key issues highlighted to ensure efficient operationalisation of interim analyses included: (a) the use of large, agile clinical trial coordinating centres/CTUs with experienced staff (particularly statisticians); (b) the detailed planning of interim analyses set out in the protocol, including all patient information sheets prepared in advance; (c) effective communication and collaborative decision-making processes across stakeholders, and effective staff training; (d) the use of electronic data capture with automated data flow processes with integrated query procedures to improve data quality; (e) the use of secure databases and data transfer procedures to maintain data integrity; and (f) prompt site query resolutions.

There were significant gaps identified in the scoping review, particularly regarding how PPIE can be best used. The currently available data suggest that whilst there is a considerable volume of evidence with regards to the conduct of adaptive trials and good trial management more generally, the current available literature offers limited specific guidance for interim analysis specifically. Whilst there are examples of innovative methods speeding up the collation and analysis of data used for interim analysis, the findings of the scoping review suggest more widespread publishing of operational opportunities and challenges would be beneficial.

5.2 Qualitative Research Findings
Nineteen participants comprising data/trial managers and statisticians, with varying levels of experience of ADs, took part in the qualitative interviews. Interviewees were primarily from the UK (n=17) but two were based in Australia. Interviews lasted between 25 and 50 minutes. While the interviews aimed to understand best practices relating to interim analysis, interviews covered many topics relating to ADs.

*Perceived advantages of ADs:* Participants agreed on the potential advantages of ADs; in particular the benefits to patients taking part in the trial who were less likely to be part of ineffective treatment arms for long durations. While ADs were perceived by many to be resource intensive, most agreed that they still offered efficiencies over non-ADs, including the speed at which they were likely to provide results.

*Interconnectivity of trial processes:* While some suggested that principles for running an AD study should not differ from any other well-managed clinical trials, many with experience of ADs described major differences in the demands placed on staff working on ADs. While the focus of interviews was on interim analyses, participants emphasised interconnected trial processes meaning that wider design considerations were fundamental to supporting fast, robust interim analysis. These included ensuring wide involvement at the design phase, streamlined communication processes, timely return of high-quality data and training at sites. Specific steps from an analysis perspective focused on advanced preparation of statistical analysis plans, simulations and code ready to go. Appropriate levels of resource were viewed as crucial as ADs were described as having the potential to place unacceptable workloads on individuals. The importance of meaningful PPIE was emphasised with some suggesting that, although potentially complex, more could be done to involve PPIE partners at key stages such as decision-making following interim analyses.

*The need for wider change:* Alongside design considerations, the need for wider system change to support the conduct of high-quality ADs was discussed. This included the need to raise funder awareness of specifics of ADs such as levels of resource and trial timelines. Emphasis was placed on the need for those with regulatory and governance responsibility to grasp principles of study conduct. Greater awareness of ADs in clinical communities was believed by some to be important. Upskilling of trial staff may be important in ensuring that those with the necessary skills are available to conduct ADs. The expansion of a body of literature to inform trial processes was also believed to be important to support increased future utilisation of ADs.

5.3 Patient and Public Involvement

Members of the PAG joined the stakeholder meeting and shared insight on the evidence synthesis and qualitative data. They noted a lack of detail about practically embedding PPIE in the interim analysis process (including in early planning) and suggested that more research is needed around approaches to PPIE when changes follow from interim analyses in adaptive trials.

Although there were no clear answers from the project findings on how PPIE should fit into high-quality, speedy, acceptable interim analyses, the PAG highlighted data that underlined the importance of accountability and transparency, and challenges around communicating a complex method and working to a changeable timeline. They suggested that working closely with a PPIE group can support future trial teams to embed accountability and transparency, and to address the challenges of communicating the method and supporting participants through a changeable timeline.

The group co-produced, with the PPIE Lead, a guidance document [Appendix 1] for future research teams; this focuses on communicating with trial participants and working with a PPIE group to ensure acceptability of interim analyses. The advice covers topics such as sensitive language, practical tools to support research delivery staff, creative activities to better understand participants’ perspectives and key considerations when involving patients and the public.

5.4 Key Recommendations

At the stakeholder meeting, participants discussed the research findings in small groups and came up with some key recommendations that would form the basis of a subsequent guidance paper.

A major point of concern was difficulty in creating guidance that would be applicable to different types of trials. For example, commercial trials, with greater resources, might not benefit from the same guidance as academic trials.

The consensus was that interim analyses should be thought of as a process rather than a single event. At the design stage, it was recommended that examples of individual simulated interim analyses be made available to key stakeholders, together with what the design would recommend for trial adaptation. This would allow discussion of potential disagreements that may arise in the future during an actual interim analysis that would otherwise cause delays.

The small groups all acknowledged the importance of resources and staffing, noting that site costs must be accurately included in budgets and plans. Early engagement with site staff may translate into quicker implementation of adaptations. More opportunities for new staff to shadow experienced staff across disciplines would be useful for capacity building.

Data cleaning was repeatedly highlighted, with emphasis on ongoing processes and the use of tools like Data Validation Plans to prioritise important data fields for interim analysis. It was suggested that methods used for conducting interim analyses be described in detail when writing up adaptive trials to fill gaps in the literature. Having a full dry run of an interim analysis in advance of the actual first one would allow issues that could cause delays to be identified in advance and addressed.

Finally, it was highlighted that there was a need for a practical guide on managing interim analyses, including checklists for data checks, Gantt charts for scheduling, and the potential for mentorship to provide practical, real-world advice.

5.5 Discussion and Limitations

The ROBIN project used several research methodologies to identify current best practice for conducting high-quality and speedy interim analyses for adaptive clinical trials. The evidence synthesis and qualitative research were conducted according to high standards, and useful supplemental insights were added by an experienced PAG. A limitation of the project is that we had only one wider stakeholder meeting and may have produced more robust recommendations and guidance had we adopted a multi-step procedure to promote consensus and reflection.

Although we identified some current research and best practice, there were fewer tools and well-described procedures identified than we had anticipated. We would encourage more adaptive trial teams to publish papers that highlight reproducible procedures that they implemented to ensure high-quality and speedy interim analyses, as well as learnings from studies that experienced unanticipated challenges.

Another limitation is that our qualitative research and stakeholder engagement was focused on UK academic trials. It may be that the pharmaceutical industry and academic groups in different countries are developing their own best practices in adaptive trials that we did not discover.

*5.6 Conclusions*

We have advanced the understanding of best practices for conducting interim analyses in adaptive trials through synthesising current publications and conducting in-depth interviews with stakeholders. This project lays the groundwork for more efficient implementation of interim analyses. Future research that aims to integrate robust PPIE and detailed operational procedures will be crucial to continue enhancing the benefit provided by adaptive clinical trials.

1. **Dissemination**

We are currently drafting two papers that respectively report the evidence synthesis and qualitative research findings. We will then develop a paper that summarises the overall findings and provides more detailed recommendations for implementing interim analyses for ADs. These papers will all be made open-access.

Several abstracts have been submitted to the 2024 International Clinical Trials Methodology Conference. Short summaries of the papers and presentations will be prepared and disseminated via social media. All project outputs will be linked to in the PANDA repository (<https://panda.shef.ac.uk/>). Initial findings of the project were presented at the 2024 Adaptive Designs and Multiple Testing Procedures conference in Ibiza (https://admtp.github.io/ADMTP2024/).

We will work closely with the UKCRC, NIHR and the TMRP, particularly the Adaptive Designs Working Group, to disseminate the results and take forward the highlighted methodology gaps for further grant applications.

**Word count** (sections 1-6) (limit 3000 words)

1. **Acknowledgements**

Leadership and coordination**:**

James Wason, Professor of Biostatistics, Newcastle University

Dawn Teare, Professor of Biostatistics, Newcastle University

Helen Hancock, Professor of Clinical Trials, Newcastle University

Project Management Group:

Opeyemi Agbeleye, Research Assistant, Newcastle University

Michelle Bardgett, Senior Trial Manager, Newcastle University

Alex Bevin-Nicholls, PPIE Manager, Newcastle University

Dawn Craig, Professor in Evidence Synthesis, Newcastle University

Matt Breckons, Senior Research Associate, Newcastle University

Mike Cole, Research Associate, Newcastle University

Helen Mossop, Senior Research Associate, Newcastle University

Chizoba Oparah, Research Assistant, Newcastle University

Julia Phillipson, Data Manager, Newcastle University

Katie Thomson, Senior Research Associate, Newcastle University

Zoe Walmsley, Trial Manager, Newcastle University

Nina Wilson, Senior Research Associate, Newcastle University

Other co-authors:

Munya Dimairo, Senior Research Fellow, School of Medicine and Population Health, University of Sheffield

Martin Law, Research Associate, MRC Biostatistics Unit, University of Cambridge, Royal Papworth Hospital NHS Foundation Trust, Cambridge

Nurulamin Noor, Academic Clinical Lecturer, University of Cambridge

Philip Pallmann, Senior Research Fellow, Centre for Trials Research, Cardiff University

David Robertson, Senior Research Associate, MRC Biostatistics Unit, University of Cambridge

Christina Yap, Clinical Trials and Statistics Unit, The Institute of Cancer Research

**Funding acknowledgement and Disclaimer:**

This project was funded by the National Institute for Health Research (NIHR) CTU Support Funding scheme. The views expressed are those of the author(s) and not necessarily those of the NIHR or the Department of Health and Social Care.

1. **References**

1. DiMasi JA, Grabowski HG, Hansen RW. Innovation in the pharmaceutical industry: New estimates of R&D costs. Journal of Health Economics. 2016;47:20–33.

2. Bentley C, Cressman S, van der Hoek K, Arts K, Dancey J, Peacock S. Conducting clinical trials—costs, impacts, and the value of clinical trials networks: A scoping review. Clinical Trials. 2019 Apr 1;16(2):183–93.

3. Pallmann P, Bedding AW, Choodari-Oskooei B, Dimairo M, Flight L, Hampson LV, et al. Adaptive designs in clinical trials: why use them, and how to run and report them. BMC Medicine. 2018;16(1):29.

4. Burnett T, Mozgunov P, Pallmann P, Villar SS, Wheeler GM, Jaki T. Adding flexibility to clinical trial designs: an example-based guide to the practical use of adaptive designs. BMC Medicine. 2020 Nov 19;18(1):352.

5. Bothwell LE, Avorn J, Khan NF, Kesselheim AS. Adaptive design clinical trials: a review of the literature and ClinicalTrials.gov. BMJ open. 2018;8(2):e018320.

6. Noor NM, Love SB, Isaacs T, Kaplan R, Parmar MKB, Sydes MR. Uptake of the multi-arm multi-stage (MAMS) adaptive platform approach: a trial-registry review of late-phase randomised clinical trials. BMJ Open. 2022 Mar;12(3):e055615.

7. Stallard N, Hampson L, Benda N, Brannath W, Burnett T, Friede T, et al. Efficient Adaptive Designs for Clinical Trials of Interventions for COVID-19. null. 2020 Oct 1;12(4):483–97.

8. The Adaptive Platform Trials Coalition. Adaptive platform trials: definition, design, conduct and reporting considerations. Nat Rev Drug Discov. 2019 Oct;18(10):797–807.

9. Wason JMS, Brocklehurst P, Yap C. When to keep it simple – adaptive designs are not always useful. BMC Medicine. 2019 Aug 2;17(1):152.

10. Mukherjee A, Wason JMS, Grayling MJ. When is a two-stage single-arm trial efficient? An evaluation of the impact of outcome delay. European Journal of Cancer. 2022 May;166:270–8.

1. **Appendices**

Appendix 1: ROBIN PPI report

1. **Conflict of interest declaration**

There were no competing interests relevant to this work.
