# Supplementary material for: Practical guidance for conducting high-quality and rapid interim analyses in adaptive clinical trials
Source: BMC Med. 2025 Oct 1;23:528. doi: 10.1186/s12916-025-04362-x (PMC12487222; doi:10.1186/s12916-025-04362-x)
Supplement: Supplementary file 2 — Supplementary Material 2: ROBust INterims for adaptive designs (ROBIN): Patient and Public Involvement (PPI) Report. [file 12916_2025_4362_MOESM2_ESM.docx]

*
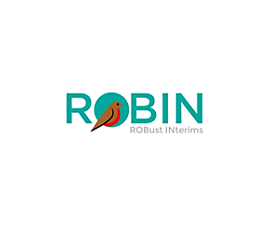
***ROBust INterims for adaptive designs (ROBIN):**

**Patient and Public Involvement (PPI) Report**

**Report authors:** Jim Ainslie (public contributor), Alex Bevin-Nicholls (PPI Lead), Michal Chantkowski (public contributor), Jenny Earle (public contributor), Caroline Kemp (public contributor), Rosemary Nicholls (public contributor), John Robson (public contributor), Louise Thomson (public contributor).

A group of 7 public contributors kindly shared their time, insight and feedback, to support the ROBust INterims for adaptive designs (ROBIN) project. This Public Advisory Group (PAG) gave guidance to the research team during the design and delivery of the project, and ensured that we focused on not only the quality and speed of interim analyses, but also, crucially, their acceptability from a public perspective.

We are hugely grateful to members of the PAG for their input. The following report summarises their contributions and advice for future research teams, around two key themes:

- **Communicating with trial participants**, and considering the emotional impact on them if parts of the trial change;
- **Working with a Patient and Public Involvement (PPI) group** to ensure the interim analysis processes and any resulting changes are acceptable from the patient and public perspective.

**Stakeholder meeting in Leeds to discuss project results & guidance, November 2023**

At the meeting, some key points (below) were presented and discussed from the ROBIN evidence synthesis and interview data, some of which explicitly relate to patient and public involvement (PPI) and some of which the ROBIN PAG felt had an implicit link to planning/facilitating PPI:

- There was some acknowledgement and agreement among interviewed trial staff that PPI should be embedded in the interim analysis process;
- There is relatively little detail in the published literature discussing how PPI can be embedded in this process;
- More information and, we suggest, more research is needed on approaches to PPI when planning and doing interim analyses in adaptive design trials;
- The data highlighted that adequate staff resource and budget are needed to do interim analyses well – we propose that adequate resources are key for PPI too;
- There were some identified challenges around the logistics of decision-making in the interim analysis process, e.g. organising meetings without delay, and it is important to plan PPI into this process to avoid unnecessary delays;
- Staff may experience an increased workload and pressure when working on trials with adaptive designs and changes as a result of interim analyses – the ROBIN PAG identified this as an important consideration to them, which should be considered in discussions around the benefits and drawbacks of adaptive designs, and considered when planning and doing interim analyses, to protect staff mental health;
- There were no clear answers from the data on how PPI should fit into high quality, speedy, acceptable interim analyses, but there was some acknowledgement that accountability and transparency are important, and that challenges exist around communicating a complex method and working to a changeable timeline. The ROBIN PAG suggest that working closely with a PPI group can support future research teams to embed accountability and transparency in their processes, and to address the challenges of communicating the method and supporting participants through a changeable timeline.

**Guidance from the ROBIN PAG on the themes of communicating with trial participants and working with a PPI group to ensure acceptability of interim analyses**

**Advice on communicating with trial participants:**

- **How much should a research team tell participants about the trial design and interim analyses? What’s the most appropriate level of detail to give? This can be discussed with a trial’s PPI group.**

I think the important thing here is that the staff involved in communicating with the participants are fully briefed so that they can answer all questions that are asked by those participants that do wish to know more.

- **Clarity, sensitivity, and mode of communication are all important and research teams should consider who is communicating with participants, and how can they be supported to do this well:**

Communication will be key, and sensitive use of language.

- **Discussion with a PPI group will help research teams to think through communication with participants.** Teams should be aware of power imbalances between ‘researcher’ and ‘researched’, and that this can be increased when someone is experiencing ill health, too. Discussing plans and communication with a PPI group can help the research team to understand more from a patient perspective.

You need to understand participants’ needs and fears. This is where good PPI would help.

- **Remember to consider the role of family/carers/friends in supporting research participants –they might find information about the trial, and the potential changes after interim analyses, useful too.**
- **Researchers should consider signposting and support for people leaving the trial, if the arm that they are allocated to is stopped after interim analyses.**

A good exit strategy is needed. I always like to be given some other options. This is where managing expectations is crucial. At the outset, this needs careful planning and consideration.

Can participants who are no longer involved due to changes during an adaptive trial be signposted to other similar or related trials?

- **Researchers should communicate with transparency about the potential benefits of adaptive trial designs and keep in mind that participants may have different reactions to the idea of a trial changing in any way, part-way through (i.e. some people may have concerns).**

Be aware that there will be a range of individual patient responses to the idea of stopping a trial midway. Some may take it in their stride, even be pleased that they don’t need to continue with what they’ve been prescribed. Others may feel distressed at the thought that they’ve been part of a failed experiment.

Side effects of drugs can be long-term even after use of the drug has been discontinued or arrive after more than 9 months. Maybe that applies to their benefits too? I’d suggest speed of research is not only an advantage.

- **Although all trials are conducted based on uncertainty – because researchers don’t know if one treatment is better than another – the idea of changes after interim analyses may introduce an extra layer of uncertainty to participants, that they may find unnerving, and this should be addressed in information shared from the outset.**

Some participants may have an albeit unreasonable conviction that the Doctor is all-knowing and feel challenged by the thought that he/she is asking them to take medicine that he/she has doubts about. How this is explained at the outset needs preparation and care to ensure consent.

- **Research teams should remember the crucial principle of doing no harm (including emotionally) and be prepared to address participants’ concerns and answer their questions, which may be focused around the points of change in the trial – being well-prepared for these reactions and questions may help to increase the likelihood of the patient joining the trial in the first place, staying enrolled in it (where possible) through periods of change, and taking part in other research projects in future. It may also help to convey potential benefits of the trial design.**

How the information that, for example, a trial arm is stopping, is conveyed to participants involved in it could have an impact on their wellbeing and willingness to take part in a trial in the future. They may ask:

1. How do you know the treatment isn’t effective before the trial ends?
2. Are there going to be any long-term effects on me, having taken this ‘failed’ treatment?
3. Have I been a guinea pig?
4. What happens to me now?
5. Is saving money the reason for stopping my trial?
6. If I feel my condition has improved, but my trial arm is stopped, will I still be able to receive the medication that I was given?

Could a list of possible questions and answers be a useful tool for research nurses or other trial delivery staff to have, as they have direct contact with participants but may not have been involved in planning adaptive design and have answers ready for all questions about it?

I think it is important for the contact staff to be able to respond sensitively to these questions, but also to convey the benefits of being on a trial with interim analysis. Managing expectations is key, but also outlining the potential positives for the participants now and for patients in the future.

- **Role play could be a useful tool to help researchers and trial teams understand how a participant might feel, and therefore what their information and support needs might be (and how to address concerns and questions), when they are enrolled on a trial that has potential changes in its pathway.**

I loved the suggestion to involve researchers in role play. I think using scenarios from the participant point of view could be helpful in many ways: patient information, dissemination, recruitment of PPI members etc.

Performing a five-minute role play with ‘the clinician’ adopting contrasting approaches to informing ‘the patient’ that the trial arm is to be changed could be very informative and an effective training method.

- **The ROBIN PAG feel that burden on staff delivering a trial is an important consideration, to both protect staff mental health and to ensure time and energy for effective communication with participants on the trial.**

What does concern me is the burden on staff to analyse large sets of complex data in a short time or shorter time and it also to be high quality. We have to support staff and do them no harm - how this is planned for them to deliver is crucial. If staff are stressed and anxious and ill equipped and poorly prepared, it won’t pan out well.

**Advice on working with a Patient and Public Involvement (PPI) group to ensure interim analysis processes and resulting changes are acceptable to participants:**

- **A PPI group or contributors should be involved in designing the trial, to make sure that patients and the public help researchers to decide which criteria and data any interim analyses will be based on, and make shared decisions around what might be important outcomes to focus on.**

Remember that a doctor might want to see a reduced blood pressure reading, as a measure of success of a treatment, while a trial participant might want to be able to walk to their friend’s house.

- **Making sure that interim analyses (and any resulting changes to a trial) are acceptable to participants is a key discussion point for early PPI, and is a foundation for successful adaptive design trials – if we focus too much on speed and robustness of interim analyses, and forget about acceptability, then we risk research that is irrelevant at best and unethical at worst.**
- **Researchers should produce a timeline or outline of an example participant journey through the trial, including around any interim analysis points, so that the trial PPI group have a clear idea of how a participant would experience the trial, and can therefore share their views on making this experience as accessible and inclusive as possible.**

I like a map of the journey. The PPI in this could help with the planning, the language, how best to do it and what support is needed. This might include advising on accessibility of technology, financial considerations for travel or out of pocket expenses, or importantly also emotional aspects.

- **A PPI group for an adaptive design trial should include public contributors with different familiarity with this type of trial. People with no familiarity can ask ‘outsider questions’ to help strengthen the plans for interim analysis.**
- **The PPI group should bring together people with a range of views and experience, from diverse backgrounds.**

I think is good to try and recruit a diverse PPI team in terms of equity, diversity and inclusion… I think it is reaching out into communities that helps with that.

- **Support and training should be discussed with the PPI group, to find out what they would find useful and how they would feel best supported to be meaningfully involved. There should be a clear contact for questions or concerns.**
- **There should be regular feedback and communication with the PPI group, including in between meetings.**
- **More case studies about PPI in adaptive design trials should be shared to help future research teams learn from those who have already worked together – sharing both what worked well and being honest about what didn’t.**

**Anything else? Questions to consider…**

- The ROBIN PAG suggested that impact on the environment should be a consideration when planning and communicating the features and potential benefits of adaptive design trials and any change resulting from interim analyses.
- The group posed a question around what the role of Artificial Intelligence (AI) will be in data analysis in the future, and could this change how interim analyses are done in adaptive design trials?
