# Supplementary material for: Practical guidance for conducting high-quality and rapid interim analyses in adaptive clinical trials
Source: BMC Med. 2025 Oct 1;23:528. doi: 10.1186/s12916-025-04362-x (PMC12487222; doi:10.1186/s12916-025-04362-x)
Supplement: Supplementary file 3 — Supplementary Material 3: Appendix – Data Management and Validation Plans. [file 12916_2025_4362_MOESM3_ESM.docx]

**Appendix – Data Management and Validation Plans**

A Data Management Plan (DMP) is a comprehensive document which describes how trial data will be collected and handled. This would typically cover all data management activities and processes, including data collection, data processing, data storage, data extraction and data lock.

A Data Validation Plan (DVP) specifies all validation checks to be performed on trial data. This can include checks for data accuracy, data completeness and data consistency. In some cases, data accuracy checks, where trial data are compared against source data, may be documented elsewhere, for example in a Monitoring Plan.

We recommend the use of these documents and provide some components to be considered for adaptive trials designs with interim analyses. Note this is not intended as an exhaustive list of items to include in a DMP / DVP.

**Critical data items**

- State which data items, including at which timepoints, are used to define each primary and secondary outcome measure. This ensures that all parties are aware of which data items contribute to each outcome measure so data validation checks can be prioritised accordingly.
- It may also be helpful to specify data items used to define other important measures, such as treatment adherence, important covariates used for analyses, or to define analysis populations.
- See example table below

| **Outcome measure** | **Description** | **Associated data items** |
| --- | --- | --- |
| Primary outcome |  |  |
| Secondary outcome |  |  |
| Secondary outcome |  |  |
| Exploratory Outcome |  |  |
| Dose compliance |  |  |
| Disease severity |  |  |

**Data consistency checks**

- For each data validation check, in addition to specifying the details of the check to be performed, state whether the validation corresponds to a data item needed for the interim analysis and assign a priority status (e.g. low / medium / high) for the check. See example table below.

| Visit | eCRF | Question | Validation | Used for Interim Analysis | Priority |
| --- | --- | --- | --- | --- | --- |
| *[the visit(s) the data item to be checked is collect at]* | *[the eCRF the data item to be checked is collect on]* | *[the data item to be checked]* | *[specify what is to be checked]* | *[Is the data item used for interim analysis]* | *[e.g. low/medium/high]* |
|  |  |  |  |  |  |
|  |  |  |  |  |  |
|  |  |  |  |  |  |

- Specify the planned frequency for conducting validation checks, timelines for query resolution, and escalation plans if queries are not resolved in a timely manner. These could differ depending on assigned priority status.
- Specify roles and responsibilities, including who will be responsible for performing validation checks. For programmed checks, consider including which programs are used to perform each check.

**Data completion**

- Include plans for monitoring and reporting data completeness / missing data, including frequency / timing of monitoring. It is recommended this is reported for critical data items (e.g. primary outcome data) and by site.
- Specify plans to resolve unacceptable levels of missing data.

**Data accuracy**

- State which data items will be subject to data accuracy checks, the frequency / timing of these checks and the number / proportion of participants to be checked. This may instead be included in a Monitoring Plan.

**Reconciliation**

- Specify any reconciliation checks to be performed between CRF and non-CRF data, e.g. AE CRFs and SAE reports, sample tracking CRFs and non-CRF laboratory results data.

**General**

- Specify tasks to be completed prior to each interim analysis (e.g. query resolution for all high priority validations on critical data points, SAE reconciliation, AE coding, data accuracy checks for critical data items)

Further information, and a DMP template, are available at the following link: <https://acdmglobal.org/wp-content/uploads/2022/01/ACDM22-Data-Management-Plan.pdf>
