# Supplementary material for: Practical guidance for conducting high-quality and rapid interim analyses in adaptive clinical trials
Source: BMC Med. 2025 Oct 1;23:528. doi: 10.1186/s12916-025-04362-x (PMC12487222; doi:10.1186/s12916-025-04362-x)
Supplement: Supplementary file 4 — Supplementary Material 4: Role-Specific Checklists for Interim Analyses in Adaptive Trials. [file 12916_2025_4362_MOESM4_ESM.docx]

# Supplementary Material: Role-Specific Checklists for Interim Analyses in Adaptive Trials

## Trial Manager Checklist

### Planning Stage

☐ Ensure sufficient resources are allocated (e.g. in the funding application) to support adaptive design logistics.

### Trial Setup

☐ Ensure interim analysis timing(s) / frequencies and criteria for making pre-planned adaptions are pre-specified in the protocol.

☐ Develop trial protocol and Patient Information Sheet (PIS) to avoid or minimise need for amendments after interim adaptations.

☐ Engage with sponsors, regulatory agencies and ethics committees where needed to mitigate any risks associated with the adaptive design and reduce the need for repeated approvals where possible.

☐ Conduct a risk assessment for the trial, considering factors related to the adaptive design

☐ Develop a monitoring plan for the trial, detailing any requirements related to interim analyses (e.g. need for Source Data Verification prior to interim analysis).

☐ Liaise with potential sites to assess capacity for timely data entry, data query resolution, and monitoring.

☐ Plan and deliver site training sessions on adaptive design features and highlight potential implications on their role.

☐ Establish clear roles and responsibilities within the trial team for interim activities.

☐ Support the identification of oversight committee members (e.g. IDMC, TSC).

☐ Coordinate early engagement with oversight committee members and confirm availability / flexibility for potentially frequent or short-notice meetings.

☐ Develop oversight committee charters to cover aspects specific to interim analyses, for example:

☐ Whether the oversight committee will need to review all interim analyses and / or approve all adaptions

☐ Whether any interim reviews / decisions can be made by email correspondence rather than requiring a meeting.

### Before each Interim Analysis

☐ Monitor recruitment and data collection timelines to forecast when interim analysis timepoints will be reached and communicate projected timelines with relevant team members.

☐ Inform all relevant team members when an interim analysis timepoint is reached and coordinate meetings (e.g. with IDMC and TSC) as appropriate.

☐ *Optional:* Coordinate pre-interim analysis briefings with key team members to review planned processes

☐ Ensure readiness of documentation for potential adaptations.

☐ Maintain regular site communication to manage expectations around potential changes.

### During each Interim Analysis

☐ Coordinate availability of staff and systems needed to implement any adaptations.

☐ Monitor workload and reallocate resources where necessary.

### Post-Interim Analysis

☐ Update trial documentation and submit for regulatory / ethical approval as needed.

☐ Coordinate site training for any changes.

☐ *Optional:* Review feasibility and capacity at sites after adaptions are implemented if needed (e.g. if sample size increased following sample size re-estimation).

☐ Document lessons learned to inform future interim analyses and update any processes / procedures as necessary.

## Data Manager Checklist

### Planning Stage

☐ Ensure sufficient resources are allocated (e.g. in the funding application) to support data management activities associated with the adaptive design (e.g. enhanced data cleaning, need for database amendments etc.).

### Trial Setup

☐ Design CRFs / trial database to facilitate efficient capture of key variables required for interim analyses and ensure data extraction processes are flexible and reliable.

☐ Incorporate validation rules and edit checks into the trial database to generate data queries in real-time.

☐ Where relevant, ensure randomisation system has flexibility to incorporate potential changes.

☐ Participate in defining key data items and acceptable data quality metrics for interim analyses.

☐ Develop procedures for ongoing data management activities (e.g. in a Data Management Plan), including factors related to the adaptive design e.g.

☐ Risk-based data lock processes for interim analyses

☐ Data cleaning and query resolution processes for key data items, including acceptable timeframes for data entry, query resolution and any escalation processes

☐ How / when data quality metrics will be reported to the trial team for key data items

☐ Develop a Data Validation Plan detailing specific validation checks to be performed, prioritising interim-critical fields.

### Before each Interim Analysis

☐ Monitor data entry, data quality and query resolution against expected timelines and quality metrics.

☐ Maintain a continual data cleaning process, particularly for interim-critical variables, utilizing automated tools where possible.

☐ Ensure data is locked or frozen as per agreed processes prior to interim analysis.

### During each Interim Analysis

☐ Support data extraction and preparation.

☐ Assist with any urgent data issues or revalidation.

☐ Where applicable, to expedite implementation, plan for any database or randomisation system changes which may be required a result of the interim analysis decision.

### Post-Interim Analysis

☐ Implement any changes to the database or randomisation system required a result of the interim analysis decision.

☐ Update any documentation (e.g. Data Validation Plan) as appropriate following any database or randomisation system changes.

☐ Provide training for site staff on any database or randomisation system changes as needed.

☐ Resume regular data management activities.

☐ Document lessons learned to inform future interim analyses and update any processes / procedures as necessary.

## Trial Statistician Checklist

### Planning Stage

☐ Advise on features of adaptive trial design, including criteria for adaptions, timing and frequency of interim analyses.

☐ Assess the statistical properties of the proposed design (e.g. via simulations) and share with key stakeholders.

☐ *Optional:* Provide visualisation of simulated interim analyses for stakeholder discussion.

☐ Ensure sufficient resources are allocated (e.g. in the funding application) to support statistical activities associated with the adaptive design (including any need for unblinded and blinded statisticians, independent QC statistician etc.).

### Trial Setup

☐ Include details of adaptive trial design and interim analyses in the trial protocol (e.g. criteria for adaptions, timing and frequency of interim analyses, outline of analysis methods to be used, statistical properties of the design etc.).

☐ Contribute to defining key data items and acceptable data quality metrics for interim analyses.

☐ Consider whether adaptive elements could be automated during implementation (e.g. for response adaptive randomisation).

☐ Consider requirements for blinding within the statistical team and whether a separate unblinded statistician will be required for conducting interim analyses.

☐ Ensure processes and procedures are in place to restrict access to unblinded data and reports.

### Before first Interim Analysis

☐ Prepare documentation for oversight committees (e.g. IDMC), including training on adaptive design and any interim decision rules, interpretation aids, and a template interim analysis report.

☐ Document procedures for interim analyses, including roles and responsibilities of all statistical team members, Quality Control (QC) processes for interim outputs, and how access to interim unblinded data will be appropriately restricted.

☐ Develop the Statistical Analysis Plan (SAP), including details of the interim analyses and how adaptions affect the final analysis.

☐ Develop and validate code for performing interim analyses and producing (semi-) automated reports where required.

☐ Develop detailed guidance / instructions on code to be used for performing each interim analysis and how it should be run.

☐ Conduct a dry run of end-to-end interim analysis pipeline on trial data (e.g. with dummy treatment allocation data to maintain blinding) to identify any potential issues or bottlenecks.

### During each Interim Analysis

☐ Execute interim analysis as per SAP.

☐ Perform QC/peer review of outputs as per QC plan.

☐ Facilitate discussion of reports / outputs with the IDMC to support interpretation and communication.

### Post-Interim Analysis

☐ Document lessons learned to inform future interim analyses and update any documents (e.g. SAP, interim analysis code), processes / procedures as necessary.

☐ Document / report any deviations from the SAP or QC process where applicable.

☐ Archive code and outputs in accordance with SOPs.

## IDMC Member Checklist

### Trial Setup

☐ Contribute to IDMC charter and processes, including whether the IDMC will need to review all interim analyses / approve all interim adaptions.

☐ Confirm availability for interim analysis windows and flexibility for meetings.

☐ Receive training or briefing on adaptive design structure to understand criteria for interim adaptions and trial operating characteristics.

☐ Review simulated interim analyses and reporting formats, providing feedback and suggested amendments where requirement.

☐ Agree to template interim report format.

☐ Ensure understanding of responsibilities and procedures.

### Pre-Interim Analysis

☐ Review draft or simulated reports if available, requesting any changes to proposed reports well in advance of the interim analysis.

☐ Familiarise with SAP and expected decision rules.

☐ Request any additional reading or training materials as required.

### During Interim Analysis

☐ Attend IDMC meeting and review interim report.

☐ Contribute to discussion and decision on adaptation(s).

☐ Ensure recommendation aligns with both clinical and statistical considerations.

### Post-Interim Analysis

☐ Provide feedback to appropriate party (e.g. TSC/sponsor/funder) where appropriate.

☐ Ensure discussions (including and dissenting views) and decisions around adaptions are appropriately and accurately recorded in meeting minutes.

☐ Identify and feedback any process improvements for future meetings.
